# Inflammation-mediated fetal injury by maternal granulocyte-colony stimulating factor and high-dose intraamniotic endotoxin in the caprine model

**DOI:** 10.4274/tjod.tjod.galenos.2019.92300

**Published:** 2019-03-27

**Authors:** Mekin Sezik, Afşin Köker, Özlem Özmen, Mehmet Halıgür, Duygu Kaşıkçı, Ahmet Aydoğan, Orhan Özatik

**Affiliations:** 1 Süleyman Demirel University Faculty of Medicine, Department of Obstetrics and Gynecology, Isparta, Turkey; 2 Mehmet Akif Ersoy University Faculty of Veterinary Medicine, Department of Obstetrics and Gynecology, Burdur, Turkey; 3 Mehmet Akif Ersoy University Faculty of Veterinary Medicine, Department of Pathology, Burdur, Turkey; 4 Çukurova University Faculty of Ceyhan Veterinary Medicine, Department of Pathology, Adana, Turkey; 5 Isparta University of Applied Sciences Faculty of Agricultural Sciences and Technologies, Department of Animal Science, Isparta, Turkey; 6 Kütahya Health Sciences University Faculty of Medicine, Department of Histology and Embryology, Kütahya, Turkey

**Keywords:** Animal model, endotoxins, cerebral palsy, chorioamnionitis, inflammation

## Abstract

**Objective:**

To define a novel experimental model with maternal intravenous (i.v.) granulocyte-colony stimulating factor (G-CSF) followed by a single- and high-dose of 20 mg intra-amniotic (IA) endotoxin to induce fetal brain injury in the preterm fetal goat.

**Materials and Methods:**

Pregnant goats (n=4) were given 50 microg/day G-CSF into the maternal jugular vein through gestational days 110-115 (term, 150 days). At gestational day 115, 20 mg of IA endotoxin was administered. Following preterm delivery at day 120 by cesarean section umbilical cord, fetal lung and brain tissues were harvested for histopathology, immunohistochemistry, and electron microscopy. Inflammatory markers were evaluated in the amniotic fluid and fetal plasma.

**Results:**

Necrotizing funisitis with abundant leukocyte infiltration and fetal brain injury was induced in all the fetuses in the experimental group.

**Conclusion:**

Maternal i.v. G-CSF for 5 days followed by 20 mg of IA endotoxin is a feasible caprine model to exacerbate intrauterine inflammation.

**PRECIS:** Using low-dose maternal G-CSF and high-dose IA endotoxin, we have defined a feasible animal model of inflammation-mediated fetal injury in the preterm goat.

## Introduction

Fetoplacental inflammation secondary to intra-amniotic (IA) microbial colonization and subclinical chorioamnionitis provide a basis for the development of preterm birth and cerebral palsy (CP) by preferentially affecting the fetal brain tissue. Increased number of experimental and clinical work previously focused on fetoplacental inflammation and fetal brain injury. Therefore, animal models to aggravate intrauterine inflammation were often required and used^(1)^. Within this context, the preterm small ruminant (such as sheep and goat) models that exclusively used IA endotoxin were accepted as relatively suitable designs to induce fetal lung and brain injury^(2,3)^.

Granulocyte-colony stimulating factor (G-CSF) is a hemopoietic growth factor involved in the control of neutrophil numbers. G-CSF expression is increased in response to infection or injury. Therefore, a proinflammatory role of G-CSF has been suggested, secondary to increased neutrophil production and migration to the inflamed site^(4)^.

Here, we aim to define our experience with intravenous (i.v.) recombinant (G-CSF) administrations for 5 days followed by a single-dose of 20 mg of IA endotoxin to induce IA inflammation, necrotizing funisitis, and fetal brain/lung injury in the preterm goat model. Previous small ruminant models for this purpose have not previously used combined IA high-dose endotoxin and systemic G-CSF administrations^(1,2,3)^. Moreover, maternal i.v. bolus injections of G-CSF have not been utilized before. Hence, we hypothesized that our novel and relatively simple experimental model that does not require chronic maternal or fetal instrumentation would be able to produce exacerbated inflammation in utero, at least to an extent of that observed in previous experiments^(1,2,3,5,6,7)^.

## Materials and Methods

The current study includes data on preliminary experimental groups (model and controls) formed prior to the main intervention study testing the effects of pentoxifylline on fetal brain injury^(8)^. The study protocol was subject to animal ethics committee approval by Süleyman Demirel University Animal Experimentation Local Ethics Committee (approval date and no, 23.08.2011/03). Principles of laboratory animal care (NIH publication No. 86-23, revised 1985) were followed, as well as specific national laws where applicable. Eight date-mated singleton pregnant hair goats (Capra hircus) at age of 4-5 years and prepregnancy body weight of 40±5 kg were included. Term pregnancy in hair goats is around 150 days. A singleton structurally normal ongoing pregnancy and accurate fetal biometry was confirmed by ultrasonography (BCF Easi Scan, Dundalk, Ireland) at days 28, 43, 58, 73, and 88 of pregnancy. The does were sheltered in a semi-open pen and reared on pasture and/or standard food. Water and mineral salts were provided ad libitum.

At day 110 of gestation, animals were transported to the animal clinics at the Faculty of Veterinary Medicine, Mehmet Akif Ersoy University. Randomization into 2 groups was carried out: Control (group 1, n=4) and the experimental model (group 2, n=4) groups. At gestational days 110 through 115, animals in the model group were given 50 microg/day i.v. bolus injections of G-CSF (Neupogen Roche, F. Hoffmann-La Roche Ltd, Basel, Switzerland) solubilized in 2 ml of normal saline for 5 days, whereas the controls received 2 ml of i.v. normal saline only. Jugular vein was used for all i.v. administrations.

At day 115, amniocentesis was performed as defined previously^(9)^. Following sterilization of the abdomen with povidine-iodine, a 20-gauge amniocentesis needle was inserted into the amniotic cavity under ultrasound guidance, and 10 ml of amniotic fluid was sampled. Erroneous access into the allantois was excluded by the color and viscosity of the sample^(10)^. Following sampling in the model group, 20 mg of endotoxin solution (Lipopolysaccharides from Escherichia coli 055:B5, L 2880, Sigma-Aldrich, Missouri, USA) was administered intra-amniotically, using the same needle under strict ultrasound guidance. The controls received identical amount of normal saline into the amniotic cavity.

Cesarean sections were performed with minor modifications to our previously reported technique^(11,12)^. Approximately 120 h after the amniocentesis procedures (day 120) corresponding to 0.80 of gestation, preterm birth was induced by cesarean section. The does were sedated with 0.25 mg/kg xylazine (Rompun, Bayer, Germany) and given epidural anesthesia with 25 mg lidocaine and 0.016 mg epinephrine (Jetokain, Adeka, Samsun, Turkey) into the sacrococcygeal space. Local infiltrative anesthesia with 25 mg lidocaine into the presumed incision line was also used. Following a 10-cm paralumbar skin incision, uterus was opened from its dorsal curvature. Prior to amniotomy, 10 mL of amniotic fluid was aspirated with a sterile injector, followed by delivery of the neonate. Tissue samples from fetal membranes, umbilical cord, and placenta were also harvested appropriately. Postoperatively, the does received i.m. antibiotics (200 mg procain penicillin plus 250 mg dihydrostreptomycin sulfate; Diperinisol, Bayer, İstanbul, Turkey) and analgesia with i.m. metamizole sodium (Novalgin ampoule, PharmaVision, Istanbul, Turkey).

Following drying and weighing, the kids were given 50 mg/kg intraperitoneal sodium thiopental (Pental Sodyum, IE Ulugay, Istanbul, Turkey) for euthanasia^(9,11)^. Then, intracardiac blood was sampled via the transthoracic route and chest opened followed by en bloc dissection of the lungs and brain^(11)^. Pulmonary parenchyma and cerebral white matter were sampled.

For routine histopathology and immunohistochemistry (IHC), tissue samples were fixed in 10% buffered formaldehyde and embedded into paraffin with routine processing for further histopathological and immunohistochemical staining. For transmission electron microscopy (TEM), tissue samples were carefully sliced into approximately 1-mm^3^ pieces on a petri dish that contained buffered glutaraldehyde (2.5%, pH, 7.2) as a fixative. Sliced tissues were transferred into fixative-containing dark-colored bottles, kept at 4 °C for 24 h and transported to the TEM laboratory.

For IHC evaluations fetal lung samples were stained with surfactant proteins A, B, C, and D (Santa Cruz Biotechnology Inc, USA), prosurfactant protein B (Abcam, UK), interleukin-1, interleukin-4, interleukin-6, interleukin-10, tumor necrosis factor (TNF)-alpha, caspase 3, caspase 5, caspase 7, COX-1, COX-2, interferon-alpha, and interferon-beta (Abcam, UK).

Fetal brain samples were immunostained with interleukin-1, interleukin-4, interleukin-6, interleukin-10, TNF- alpha, caspase 3, caspase 5, caspase 7, COX-1, COX-2, interferon-alpha and interferon-beta, neuron specific enolase (NSE), glial fibrillary acidic protein (GFAP) (Abcam, UK), apoptosis protease activating factor (Biosensis, Australia), vimentin (Abcam, UK), anti-neurofilament protein (NFP) (Abcam, UK), and anti-myelin basic protein (MBP) (Abcam, UK).

Placentae and fetal membranes were stained for interleukin-1, interleukin-4, interleukin-6, interleukin-10, TNF-alpha, caspase 3, caspase 5, caspase 7, COX-1, COX-2, interferon-alpha, and interferon-beta. Commercial kits were used for IHC examinations, using a routine streptavidine-biotin peroxidase technique.

To evaluate the severity of the IHC reactions semiquantitative analyses were performed, using an arbitrary visual scale with a grading score ranging from 0 to 3 as follows: (0) = negative, (1) = weak staining, (2) = moderate staining, and (3) = diffuse staining. Olympus CX41 light microscope and the Database Manual Cell Sens Life Science Imaging Software System (Olympus Corporation, Tokyo, Japan) were used for examinations. The pathologists were blinded to the experimental groups.

Samples were bleached in propylene oxide and placed into 1/1 propylene-oxidized araldehyde for 2 h and soaked in pure araldehyde overnight. Afterwards, the specimens were embedded in araldehyde and polymerized at 60 ˚C for 48 h. The 700 nm sections were cut at ultra-microtome (Leica Ultracut R, Leica Microsystem, Austria), and stained with toluidine blue and examined by light microscope (Olympus BX50, Olympus, Tokyo, Japan). Selected areas were trimmed and 60-nm thin sections were stained with uranyl acetate and lead citrate, and examined by JEOL JEM1220 Transmission Electron Microscope (Nippon Denshi Co, Tokyo, Japan).

On lung sections, ultra-structural changes such as increase in goblet cells, secretory components of goblet cells, endoplasmic reticulum in goblet cells, protein synthesis, cilial degenerations, neutrophil, lymphocyte and plasma cells infiltrations, increase in intracellular ribosomes, and thickness of noncellular basal membranes were examined. In brain tissues, increase in polymorphonuclear leukocyte in perivascular areas and demyelination were evaluated.

Interleukin and TNF-alpha levels in plasma and amniotic fluid samples were evaluated by the double antibody sandwich enzyme-linked immunosorbent assay (ELISA) method. Commercial kits for goat serum provided from Eastbiopharm (Hangzhou, China) were used for TNF-alpha, interleukin-1, interleukin-4, interleukin-6, and interleukin-10. Results were evaluated at 450 nm, and optic density (OD) values were calculated and standardized accordingly.

### Statistical Analysis

Variables were expressed as median and interquartile ranges given within brackets. Mann-Whitney U test was used for comparisons across the two groups. Wilcoxon signed-rank test was used to compare amniotic fluid measurements (at day 115 and 120) within the groups A two-sided p<0.05 was considered as significant for all analyses.

## Results

**Figure 1:**
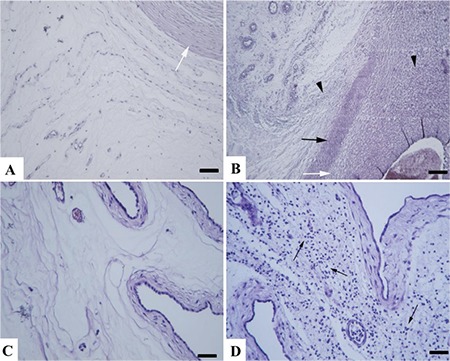
Umbilical cord and fetal membrane histopathology. (A) Normal appearance of umbilical cord from a kid in the control group, umbilical vessel (white arrow) (B) Severe funisitis characterized by inflammatory cell infiltrations (arrow heads) and necrotic arcs (black arrow) of inflammatory debris around the umbilical vessel (white arrow) and neovascularization (upper left side of the picture) from the model group. (C) Histology of normal fetal membranes from the control group. (D) Inflammation of fetal membranes showing numerous inflammatory cells (arrows) from model group, HE, Bar=100 μm

**Figure 2:**
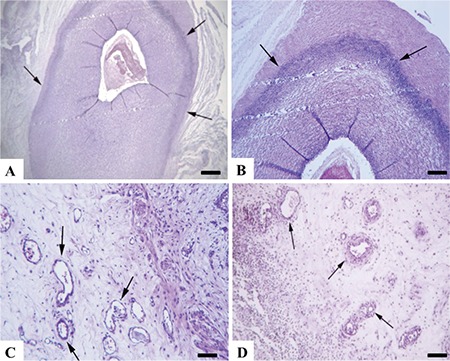
Umbilical vessel histopathology. (A) Marked necrotic arc (arrows) in vascular wall of the umbilical vessel, Bar=200 μm. (B) Higher magnification of the necrotic arc, Bar=100 μm. (C and D) Neovascularization (arrows) in umbilical cord, HE, Bars=100 μm

Macroscopically, the umbilical cords from group 1 (controls) were normal, whereas hemorrhage and edema were evident in the model group. Microscopically, funisitis and vasculitis characterized by extensive inflammatory reaction including the Wharton jelly were present in endotoxin-exposed animals. This was characterized by necrotic arcs of inflammatory debris around all vessels made up of degenerated neutrophils in the Wharton’s jelly, showing areas of inflammatory debris and neovascularization. Most of the infiltrating cells were neutrophils, while lymphocytes and plasma cells were also observed. Thrombi were observed in some of the umbilical vessel sections. Fetal membranes from group 1 were normal, whereas extensive inflammatory reaction and desquamation at the epithelial cells with areas of hemorrhage were present in membranes from the model animals. In some specimens, aggregates of bacteria presumably due to cervical dilatation secondary to inflammation in fetal membranes specific to goats or contamination were also evident (Figure 1, Figure 2).

Pulmonary sections from group 1 revealed thin septal walls, concordant with normal preterm lungs. G-CSF plus endotoxin-exposed kids had thickened and edematous septal walls accompanied by increased alveolar macrophages and neutrophilic infiltrations. Routine histopathology did not reveal any distinctive findings in the brain tissues except hyperemia and mild gliosis in a kid from group 2.

IHC results were in parallel with histopathology. Comparisons of the staining intensities across the groups are given in Table 1, revealing significant differences for all the studied parameters. Inflammatory markers including various interleukins were increased in all tissues. Moreover, specimens from the model group generally stained heavily for the apoptosis markers. Fetal brain injury was apparent in the model animals, shown by decreased NSE, NFP, GFP, and MBP staining compared to controls (Table 1).

**Figure 3:**
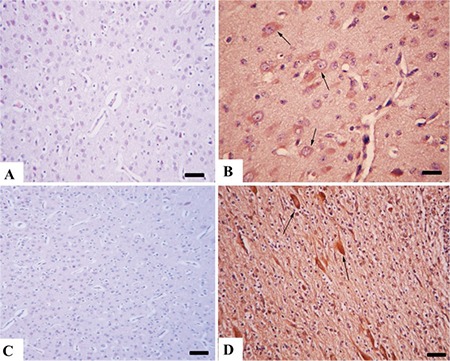
Brain immunohistochemistry. (A) Negative caspase-5 immunoreaction in the control group, Bar=100 μm. (B) Increased caspase-5 immunoreaction in neurons (arrows) from the model group, Bar=50 μm. (C) Negative COX-1 immunoreaction in the control group, Bar=100 μm. (D) Marked COX-1 reaction in the model group, Bar=100 μm

**Figure 4:**
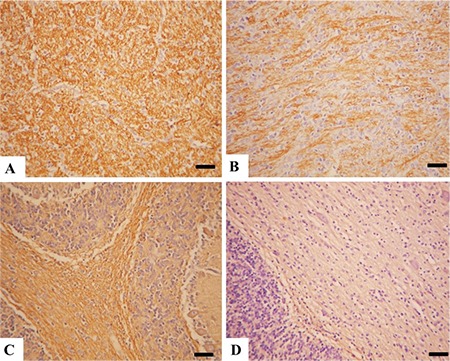
Brain tissue markers immunohistochemistry. (A) Marked myelin basic protein immunoreaction in a control brain, Bar=100 μm. (B) Decreased myelin basic protein expiration in the model group, Bar=100 μm. (C) Marked neurofilament protein immunopositive reaction in the control group, Bar=100 μm. (D) Markedly reduced neurofilament protein expiration in the model group, Bar=100 μm, streptavidin biotin peroxidase method

**Figure 5:**
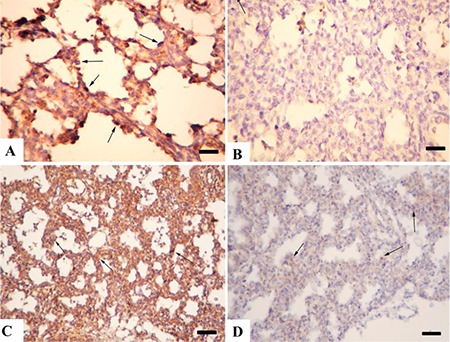
Lung immunohistochemistry (A) Strong SP-A expiration from alveolar cells (arrows) in a lung from the control group, Bar=100 μm. (B) Decreased SP-A expiration from alveolar cells (arrows) in the model group, Bar=100 μm. (C) Marked SP-B reaction in lung epithelial cells (arrows) in the control group, Bar=200μm. (D) Decreased SP-B immunoreaction in alveolar cells in lungs from the model group, Bar=100 μm

**Figure 6:**
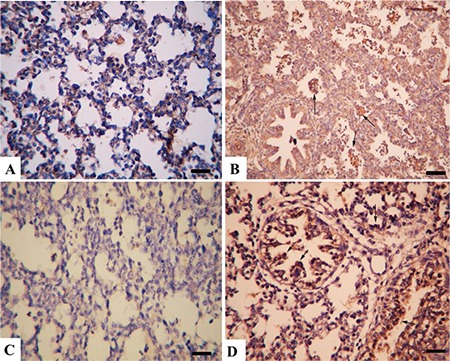
Lung interleukin (IL)-1 and caspase-5 immunohistochemistry. (A) Very slight IL-1 immunoreaction in the control group, Bar=100 μm. (B) Marked increase in IL-1 expiration from alveolar macrophages (arrows) in the model group, Bar=100 μm. (C) Negative caspase-5 expression in the control group, Bar=100 μm. (D) Increased caspase-5 immunoreaction in bronchiolar cells (arrows) in a kid’s lung from the model group, Bar=100 μm, streptavidin biotin peroxidase method

Selected IHC sections showing staining properties are shown in Figure 3 and Figure 4. Brain IHC revealed decreased immunoreaction in brain markers in the model group. While surfactant protein expirations were decreased, apoptotic markers’ expirations were increased in the lungs of endotoxin-exposed kids (Figure 5, Figure 6).

**Figure 7:**
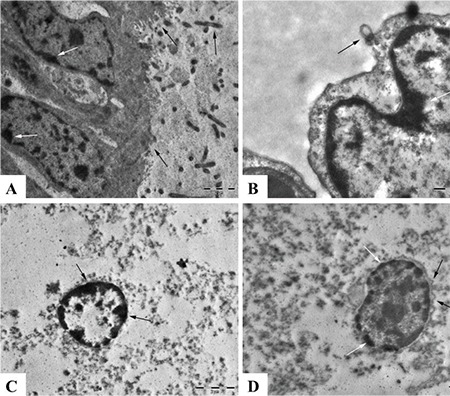
Electron microscopic findings in the model group, (A) Marked ciliary loss (black arrows) and chromatin marginations (white arrows) from lung cells, Bar=2 μm, (B) Blebbing at nuclear membrane (black arrow) and chromatin margination (white arrow) in the nucleus of a lung cell, Bar=1μm, (C) Severe necrotic changes, organelle lysis and chromatin marginations (arrows) in a neuron, Bar=2 μm, (D) Blebbings at nuclear membrane (black arrows) and chromatin marginations (white arrows) in a neuron, Bar=2 μm

In the model group, brain and lungs tissues were severely affected (Figure 7). Ultrastructural examination of the brain specimens revealed chromatin margination at the nuclei accompanied by membrane damage and tissue lysis. Pulmonary specimens revealed blebbing at alveolar and bronchiolar cells. Mitochondrial damage and accompanying blebbing formations were also observed (Figure 7).

Comparisons of interleukin (IL) and TNF-alpha levels measured by ELISA in the fetal serum and amniotic fluid samples are summarized in Table 2. Pregnancies in the model group had higher mean IL-1, 4, 6, 10 and TNF-alpha in the fetal plasma and amniotic fluid samples that were retrieved at day 115 (before endotoxin administration) and at day 120 (during preterm delivery). However, comparisons of differences over time (day 120 minus day 115) across the groups revealed no significant differences (Table 2).

IL-1, IL-4, IL-6, IL-10, and TNF-alpha levels showed non-significant increments both in the control (p=0.141, p=0.461, p=0.066, p=0.066 and p=0.066, respectively) and the study groups (p=0.461, p=0.461, p=0.461, p=0.18 and p=0.066, respectively).

## Discussion

According to our findings, the use of maternal G-CSF without fetal catheterization followed by high-dose IA endotoxin is effective to induce fetal funisitis as well as fetal brain and lung injury. The suggested model can be used in experimental research to test the efficacy of potential drugs for the prevention of inflammation-related preterm labor.

Certain models to induce subchronic chorioamnionitis associated with preterm delivery have been used in the medical literature. The most common animal model includes the injection of endotoxin, i.e. lipopolysaccharides (LPS) usually isolated from *E. coli*^(1,2,3,5,6,7,13)^. Although endotoxin has been injected into the maternal peritoneum^(14)^ or directly into the uterine horns^(15)^ of small animals such as rat and rabbit, IA administration generates one of the most plausible models, similar to the human disorder. Therefore, many experimental models used small ruminant models such as pregnant sheep and goat to induce chorioamnionitis and subsequent fetal injury by injecting endotoxin into the amniotic fluid under ultrasound guidance^(13)^. Fetal administrations of IA endotoxin to induce inflammation are technically difficult in smaller animals, but are feasible in the pregnant sheep and goat. Moreover, small ruminant models are convenient for evaluating novel fetal therapy modalities against fetal injury, as tissue harvesting and adequate sampling of amniotic fluid or fetal plasma are easier^(16)^.

Ureoplasma species, especially Ureoplasma pavum serovars of up to 2x10^7^ Colony Forming Units have also been used to induce chorioamnionitis in pregnant sheep^(17)^. However, Ureoplasma alone seems to cause modest responses and may down-regulate LPS-induced proinflammatory cytokines^(18)^.

The endotoxin dose given into the amniotic cavity of the ewes has mostly been 10 mg LPS. However, different doses such as 1 mg, 4 mg, 20 mg, and 100 mg were also evaluated and were reported not to alter birth weight or umbilical arterial blood pH and partial carbon dioxide values relative to controls^(19)^. Moreover, even ultra-high IA doses (100 mg) were not associated with fetal deaths in the sheep model^(19)^. Interestingly, relatively low doses of IA endotoxin were associated with increased fetal pulmonary maturation (instead of injury) in some investigations^(5,6)^. It is possible that IA endotoxin stimulates lung maturation by a mechanism distinct from glucocorticoids^(19)^.

The finding that low doses of IA endotoxin may paradoxically prevent fetal lung injury led investigators to modify animal models of chorioamnionitis and fetal injury. Watanabe at al.^(7)^ suggested that fetal G-CSF pretreatment before activation in utero by an IA infusion is 100% effective to induce necrotizing funisitis. This assumption depends on the observation that preterm neonates born following chorioamnionitis had significantly higher umbilical cord G-CSF levels than those without chorioamnionitis^(20)^. Moreover, fetuses with fetal inflammatory syndrome were found to have high median fetal plasma G-CSF concentrations^(21,22)^.

It is known that low-grade systemic inflammatory response is an important component of fetal brain injury^(1)^. Therefore, pretreatment with fetal G-CSF may exacerbate inflammation in the umbilical cord and the fetus. Furthermore, necrotizing funisitis has been reported as an important risk factor for the development of chronic lung disease in the human^(23)^. Therefore, the induction of necrotizing funisitis in an experimental animal model has the potential to simulate the severe form of neonatal injury. Overall, these data^(7,20,21,22,23)^ support the notion that exacerbated inflammation by G-CSF and high-dose endotoxin causes substantial fetal lung and brain injury, as opposed to fetal pulmonary maturational or protective effects observed with low-dose IA endotoxin injections.

Depending on these previous data, we developed our model in goat pregnancy by modifying the setting described by Watanabe at al.^(7)^. These authors performed a laparotomy and hysterotomy to catheterize fetal carotid arteries chronically. At least 48 h after surgery, catheterized fetuses received daily bolus infusions of G-CSF from day 125 to day 129 of gestation. While on fetal G-CSF pretreatment, 20 mg of endotoxin was administered intra-amniotically at day 127. The investigators reported 100% success rate with the model, with all animals developing necrotizing funisitis^(7)^.

We modified the experimental model by Watanabe et al.^(7)^ and preferred giving G-CSF to the doe (mother) instead of performing chronic fetal arterial catheterization, a technically demanding intervention. G-CSF was shown to cross the placenta in previous human and animal studies^(24,25,26)^. G-CSF was measurable in the amniotic fluid and fetal plasma of pregnant mice 30 min after injection, with a peak concentration reached at 2 to 4 h. Relatively high concentrations revealed that a functional placental receptor was not essential for the transfer of G-CSF across the placenta^(25)^. Similar results were obtained in rat models, demonstrating that maternally administered G-CSF crosses the placenta and has myelopoietic effects even at low concentrations in the fetus^(26)^.

G-CSF is generally considered safe in pregnancy. Although we were not able to locate any information on potential side effects of G-CSF particularly in pregnant goats, data from other species including humans demonstrate no significant adverse effects during pregnancy^(7,27)^. In a recent report^(28)^, rates of spontaneous terminations, preterm births, maternal infections, and neonatal adverse events were similar in women with and without G-CSF therapy during pregnancy.

These results^(24,25,26,27,28)^ led us to administer G-CSF directly into the venous circulation of the does. We believe this has two advantages. First, a low-grade inflammation was induced in the maternal compartment. This conforms to the human preterm birth cascades associated with early maternal infection/inflammation. Second, G-CSF in the maternal compartment is expected to cross the placenta, obviating the need for direct fetal arterial administrations. Another modification in our design was the administration of IA endotoxin by the completion of the 5-day course of maternal G-CSF injections. We suppose this is more coherent with the fetal inflammatory response syndrome, in which low-grade preclinical systemic maternal and IA inflammation is followed by a relatively abrupt insult that leads to fetal brain injury and preterm delivery.

Inflammatory markers including interleukin-1, 4, and 6 and COX-1 and COX-2 were increased in the fetal tissues and placenta of our model pregnancies. Apoptosis in terms of caspase 3, 5, and 7 was also prominent in all those tissues. Fetal brain injury was shown in the model kids, as expressions of markers such as vimentin, NSE, NFP, GFAP, MBP, and APAF-1 were all decreased. Significant fetal inflammation was additionally proved by increased interleukin and TNF concentrations in the fetal plasma and amniotic fluid.

An interesting finding of our study was lack of significant change in certain amniotic fluid inflammatory markers between day 115 and day 120. This held true for both of the groups, suggesting that IA interleukin-1, 4, 6, 10 and TNF-alpha levels were comparable 5 days after IA endotoxin exposure. In fact, these data are in line with previous findings indicating maximally induced cytokine mRNAs within only 5 h after IA endotoxin administration and decreasing to control values by 2 days^(29)^. Therefore, our design with a sampling interval of 5 days probably did not allow detecting the early and rapid elevation of IA interleukins. 

Interleukin-10 is an anti-inflammatory cytokine, capable of inhibiting synthesis of pro-inflammatory cytokines. We found increased interleukin-10 staining in the fetal brain, fetal lung, and the placenta of exposed animals compared to controls. Interleukin-10 plasma concentrations were also higher in the model animals. Although these results seem contradictory, they support previous human and animal data, denoting increased amniotic fluid and fetal tissue levels of interleukin-10 during IA infection and/or inflammation. In a recent ovine study that used IA endotoxin^(30)^, interleukin-1, 6, 8, TNF-alpha, and interleukin-10 mRNA were all reported to be increased similar to our findings. Overall, our data support increased reflex anti-inflammatory processes in our design and the involved setting.

### Study Limitations

We did not specifically evaluate physiological parameters such as fetal heart rates and arterial or amniotic pressures, as these were not part of our original experimental design. We were not able to track changes in leukocyte count or activity during the study period in the groups. Due to ethical reasons, our design did not include groups that were given only G-CSF and given only endotoxin to observe independent effects on inflammation separately. Despite these drawbacks, our robust design proved that the present novel and customized experimental model leads to profound IA and fetal inflammation as well as fetal injury, shown repeatedly by ELISA, immunohistochemistry, and electron microscopy results. Moreover, our model consistently resulted in necrotizing funisitis, which is a predictor of chronic lung disease and impaired neurological outcome.

## Conclusion

The supposed small ruminant experimental design, which includes maternal i.v. G-CSF followed by 20 mg of IA endotoxin is a feasible model to aggravate intrauterine inflammation and fetal lung/brain injury and will have potential use for research in that specific area.

## Figures and Tables

**Table 1:**
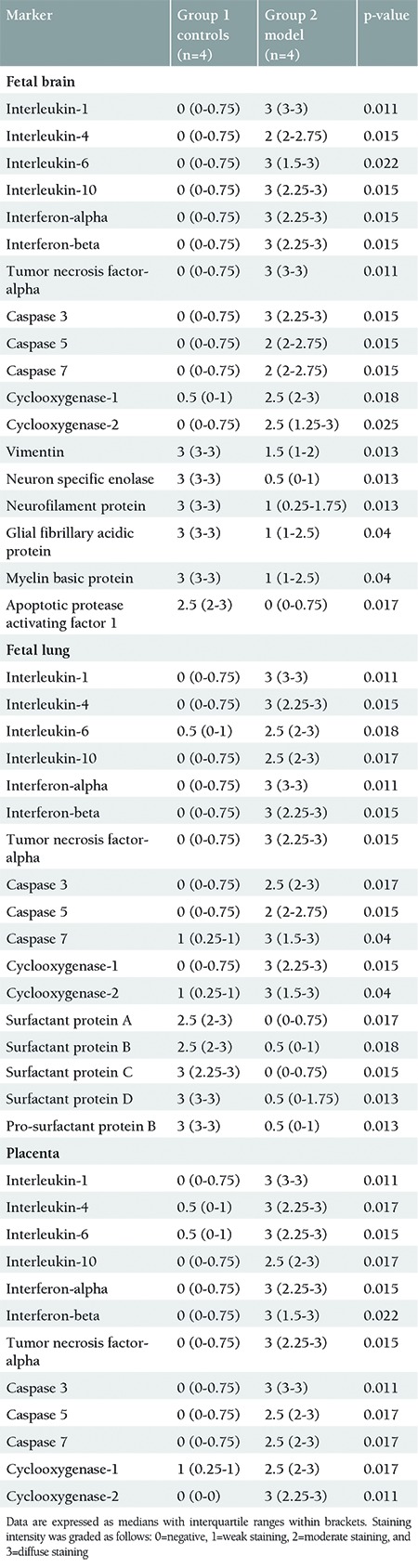
Comparisons of immunohistochemical staining intensities across the groups

**Table 2:**
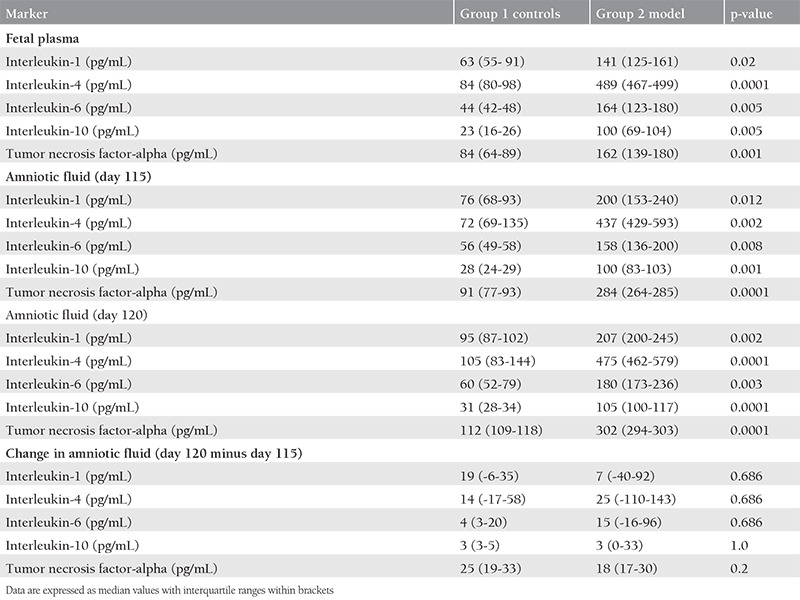
Comparisons of enzyme-linked immunosorbent assay results across the groups
